# Retraction: Study of the biological function of LncRNA LUCAT1 on cervical cancer cells by targeting miR-199b-5p

**DOI:** 10.1042/BSR-2020-0422_RET

**Published:** 2021-06-08

**Keywords:** cervical cancer, invasion, LUCAT1, miR-199b-5p, proliferation

This Retraction follows an Expression of Concern relating to this article previously published by Portland Press.

This article is being retracted from *Bioscience Reports* at the request of the Editor-in-Chief and the Editorial Board following receipt of a notification from a reader, alerting the Editorial Board to unusual repeated background features in the Western blots.

The authors have been contacted in regard to the retraction and were asked to provide the raw images for these Western blots. However as the images provided during the investigation differed from those in the published paper and appear to show signs of editing, the Editorial Board stands by the decision to retract. The authors were asked to agree to the retraction but did not respond to the Editorial Office.

